# Blinding of freeze-dried cod – a recipe developed for the FAST project

**DOI:** 10.1186/2045-7022-1-S1-P84

**Published:** 2011-08-12

**Authors:** Heidi Julius Schnoor, Marianne Witten, Ronald van Ree, Fernandez-Rivas Montserrat, Lars K Poulsen

**Affiliations:** 1 Copenhagen University Hospital, Allergy Clinic, Gentofte, Denmark; 2 AMC, Amsterdam, Netherlands; 3 Fundacion Hospital Alorcon, Madrid, Spain

## Background

The FAST project (Food Allergy Specific ImmunoTherapy) aims at the development of safe and effective treatment of food allergies. Classical allergen-specific immunotherapy (SIT) for treatment of food allergy using subcutaneous injections with food extracts has proven to be effective but too dangerous due to anaphylactic side-effects. FAST aims at developing a safe alternative by replacing food extracts with hypo-allergenic recombinant major allergens, the active ingredients of SIT. Fish allergy is caused by a single major allergen, parvalbumin. In phase I and II of the study randomized double-blind placebo-controlled trials will be performed. A recipe for fishblinding does not exist and is needed to determine the clinical reactivity when including patients in the trial and to assess efficacy in the Phase II trial.

## Double-blind placebo-controlled challenges

The aim was to develop a recipe that could blind the highest possible amount of freeze-dried cod in the lowest amount of edible low-allergenic vehicle. Several recipes were developed and tried in the attempt to hide the texture and taste of the freeze-dried cod. A burger primarily made of minced chicken meat, onion, rice and spices was able to blind cod successfully. Cumin seed is an important ingredient, that has the ability to mask the taste of cod that would otherwise remain in the mouth after intake.

## Recipe for freeze-dried cod-blinding

Freeze-dried cod powder (in active challenge)

Minced chicken meat

Onion, chopped

Olive oil

Flattened rice (rice flakes)

Thyme

Cumin seeds

Salt

Pepper

Turmeric

All ingredients are thoroughly mixed in a food processor, to a homogeneous paste. Formed to a burger and cooked in a pre-heated oven at 180°C.

7 doses are given from 50ug - 4g protein, accumulated dose of 22g of cod fish. Preliminary testing (triangle test) show that it is not possible to tell the active challenge from placebo.

